# Comparison of efficacy, safety, and quality of life between sorafenib and sunitinib as first-line therapy for Chinese patients with metastatic renal cell carcinoma

**DOI:** 10.1186/s40880-017-0230-7

**Published:** 2017-08-08

**Authors:** Wen Cai, Wen Kong, Baijun Dong, Jin Zhang, Yonghui Chen, Wei Xue, Yiran Huang, Lixin Zhou, Jiwei Huang

**Affiliations:** 1 Department of Urology, Renji Hospital, School of Medicine, Shanghai Jiao Tong University, Shanghai, 200123 P. R. China; 2 Department of Urology, Renji Hospital, School of Medicine, Shanghai Jiao Tong University, 160 Pujian Rd., Pudong District, Shanghai, 200127 P. R. China

**Keywords:** Metastatic renal cell carcinoma, Sorafenib, Sunitinib, Quality of life

## Abstract

**Background:**

Sorafenib and sunitinib are widely used as first-line targeted therapy for metastatic renal cell carcinoma (mRCC) in China. This study aimed to compare the efficacy, safety, and quality of life (QoL) in Chinese mRCC patients treated with sorafenib and sunitinib as first-line therapy.

**Methods:**

Clinical data of patients with mRCC who received sorafenib (400 mg twice daily; 4 weeks) or sunitinib (50 mg twice daily; on a schedule of 4 weeks on treatment followed by 2 weeks off) were retrieved. Primary outcomes were overall survival (OS), progression-free survival (PFS), adverse events (AEs), and QoL (SF-36 scores), and secondary outcomes were associations of clinical characteristics with QoL.

**Results:**

Medical records of 184 patients (110 in the sorafenib group and 74 in the sunitinib group) were reviewed. PFS and OS were comparable between the sorafenib and sunitinib groups (both *P* > 0.05). The occurrence rates of leukocytopenia, thrombocytopenia, and hypothyroidism were higher in the sunitinib group (36.5% vs. 10.9%, *P* < 0.001; 40.5% vs. 10.9%, *P* < 0.001; 17.6% vs. 3.6%, *P* = 0.001), and that of diarrhea was higher in the sorafenib group (62.7% vs. 35.2%, *P* < 0.001). There was no significant difference in SF-36 scores between the two groups. Multivariate analysis indicated that role-physical and bodily pain scores were associated with the occurrence rate of grade 3 or 4 AEs (*P* = 0.017 and 0.005).

**Conclusions:**

Sorafenib has comparable efficacy and lower toxicity profile than sunitinib as first-line therapy for mRCC. Both agents showed no significant impact on QoL of patients.

## Background

Molecular targeted therapy has shown promising results in different clinical trials and clinical practice, and it has been the preferred therapeutic option for patients with metastatic renal cell carcinoma (mRCC) [1–5]. Among new therapeutic agents, tyrosine kinase inhibitors (TKIs) such as sorafenib and sunitinib are used as first-line treatment agents for mRCC in China [6]. Although the efficacy of targeted therapies in terms of tumor growth control at metastatic sites is definite, their adverse events (AEs) are often major limitations. Furthermore, it is estimated that the severity of hand-foot syndrome, hypertension, diarrhea, and alopecia was higher in Japanese patients [7] than in Western patients [8, 9]. Thus, comparison of safety in clinical practice would provide important information for patient counseling and treatment decision-making.

According to the data in the Japanese study [7], it is plausible that the quality of life (QoL) of Japanese patients might have been significantly impaired with the use of TKIs. Moreover, because the Chinese ethnicity closely resembles the Japanese ethnicity, the TKI therapy-associated QoL patterns might share similarities. QoL has been a curative effect index and an important parameter to evaluate treatment efficacy in recent clinical practices. Many QoL assessment tools are available. Among them, the Chinese version of the 36-Item Short Form Health Survey Questionnaire (SF-36) has been validated and is the most commonly used tool in Chinese patients [10]. A Japanese study on QoL of patients receiving different TKIs using SF-36 reported that neither sorafenib nor sunitinib was associated with significant impairments in QoL [11].

Efficacy evaluation is important for targeted therapy. Although phase III trials compared the efficacy between pazopanib and sunitinib [12] and between axitinib and sorafenib [13], no such attempts to compare efficacy, safety, and QoL impairments simultaneously between sorafenib and sunitinib as first-line treatment for patients with mRCC have been made. Only three retrospective studies directly compared the efficacy but not QoL impairments between sorafenib and sunitinib. Choueiri et al. [5] reported that the overall response rate was higher in the sunitinib group (37%) than in the sorafenib group (9%) in two oncology centers in the US. In contrast, no significant differences in overall survival (OS) and progression-free survival (PFS) between sorafenib- and sunitinib-treated patients were observed in an Asian population (both *P* > 0.05), although sorafenib was concluded to be more favorable than sunitinib because of minimal grade 3 or 4 toxicities [14]. Recently, Sheng et al. [15] reported similar results in terms of OS and PFS of Chinese patients with mRCC (both *P* > 0.05) [15]. However, these studies were conducted with a small sample size and aimed to compare the efficacy and safety of sorafenib and sunitinib, but not the effect of these drugs on QoL.

In the present study, we retrospectively compared the efficacy, safety, and QoL impairments between sorafenib and sunitinib in Chinese patients with mRCC to further guide clinical treatment.

## Methods

### Study design and population

Data were retrieved from the electronic medical records of patients with mRCC who visited the Department of Urology in Renji Hospital affiliated to Shanghai Jiao Tong University School of Medicine (Shanghai, China) between March 2006 and July 2015. The study protocol was in accordance with the Chinese guidelines on the management of renal cell carcinoma (2015 edition) [16] and conformed to the principles of the Declaration of Helsinki.

mRCC patients who received sorafenib or sunitinib as first-line therapy with a Karnofsky performance status (KPS) score of 70–100 and completed the SF-36 questionnaire (Chinese version) at baseline and 3 months after treatment were included in the study. Informed consent was obtained from all patients. Patients who did not comply with the above criteria or had unstable or severe cardiac disease, uncontrolled brain metastases, concurrent malignancies, or incomplete data files were excluded. Personal information from medical records was anonymized and de-identified before analysis. Ethical approval was obtained from the Institutional Ethics Committee of Renji Hospital.

### QoL assessment

The filled SF-36 questionnaires were assessed independently by two authors (Wen Cai and Wen Kong), and a third person (Jiwei Huang) was consulted to resolve any disagreements.

### Clinicopathologic evaluation

Demographic information was retrieved from the medical record database. All patients underwent a pretreatment baseline evaluation including complete medical and physical examinations, complete blood count (CBC), routine organ function tests, computed tomography (CT), magnetic resonance imaging (MRI), and histological differentiation of tumor graded according to the Fuhrman’s nuclear grading system.

### Treatment

Sorafenib and sunitinib were used as first-line treatment in patients with mRCC. Sorafenib 400 mg was administered twice daily orally in a 4-week cycle, and sunitinib 50 mg was prescribed daily orally for the first 4 weeks in a 6-week cycle (4-week on/2-week off) until disease progression, intolerable AEs, or death was reported. Dose titrations were done considering the patient’s tolerance.

### Follow-up and outcome measures

All the patients were suggested to have monthly disease assessment after the treatment to estimate the treatment response and AEs. At baseline and 3 months after the treatment, the SF-36 was completed during outpatient visits. The National Cancer Institute Common Terminology Criteria for Adverse Events v3.0 [17] was used for diagnosis and grading of AEs, based on which dose titrations were carried out. The treatment was terminated in patients who experienced serious AEs, disease progression, or unacceptable toxicity (≥4-week delay in recovery to a permissible level of toxicity despite 2 dose reductions), as defined by the response evaluation criteria in solid tumors (RECIST) [18]. After treatment, patients were followed up every month until they experienced discomfort or death. PFS and OS were assessed as endpoints. PFS was defined as the duration from the onset of targeted therapy to disease progression or death as assessed by the treating physicians or the last visiting day recorded if the disease did not progress. OS was defined as the duration from the onset of targeted therapy to death or the last visiting day. The prognostic outcomes were assessed according to the Memorial Sloan-Kettering Cancer Center (MSKCC) grading model [19] and the International Metastatic Renal-Cell Carcinoma Database Consortium (IMDC) prognostic model [20].

### Statistical analyses

All analyses were made using the SAS version 9.4 (SAS Corporation, Cary, NC, USA). Categorical variables are presented as counts and percentages and were compared between the sorafenib and sunitinib groups with the Pearson Chi squared test or the Fisher exact test as appropriate. The Kaplan–Meier method was used to estimate survival curves, and the log-rank test to compare PFS and OS between the sorafenib and sunitinib groups. Patients alive at the end of the study were censored at the last follow-up. SF-36 scores are presented as mean ± standard deviation (SD) and were compared using the unpaired *t* test. The means of individual scores were defined as cutoff points. Forward stepwise logistic regression analysis was used to determine associations between clinical characteristics and SF-36 scores. All statistical tests were 2-sided, and *P* < 0.05 was considered significant.

## Results

### Clinicopathologic characteristics

A total of 184 patients, with a median age of 60 years (range 24–83 years), were selected, and 141 of them were men. Of them, 110 received sorafenib and 74 received sunitinib as first-line treatment. No significant differences in baseline clinical characteristics were found between the two groups (Table 1). Fifteen (13.6%) patients in the sorafenib group and 10 (13.5%) in the sunitinib group received second-line targeted therapy due to disease progression.

**Table 1:** Baseline clinicopathologic and prognostic characteristics of 184 patients with metastatic renal cell carcinoma (mRCC)

Variable	Total [cases (%)]	Sorafenib group [cases (%)]	Sunitinib group [cases (%)]	*P* value
Total	184	110	74	
Sex				0.336
Man	141 (76.6)	87 (84.3)	54 (73.0)	
Woman	43 (23.4)	23 (25.7)	20 (27.0)	
Age (years)				0.152
<65	139 (75.5)	79 (71.8)	60 (81.1)	
≥65	45 (24.5)	31 (28.2)	14 (18.9)	
Histology				0.872
Clear cell	176 (95.7)	105 (95.5)	71 (96.0)	
Others	8 (4.3)	5 (4.5)	3 (4.0)	
Prior nephrectomy				0.516
Yes	150 (81.5)	88 (80.0)	62 (83.8)	
No	34 (18.5)	22 (20.0)	12 (16.2)	
Prior cytokine therapy				0.118
Yes	60 (32.6)	31 (28.2)	29 (39.2)	
No	124 (67.4)	79 (71.8)	45 (60.8)	
Fuhrman grade				0.636
1–2	106 (57.6)	64 (58.2)	42 (56.8)	
3–4	64 (34.8)	35 (31.8)	29 (39.2)	
Unknown	14 (7.6)	11 (10.0)	3 (4.0)	
Number of metastatic sites				0.084
1	134 (72.8)	75 (68.2)	59 (79.7)	
≥2	50 (27.2)	35 (31.8)	15 (20.3)	
Metastatic sites				
Lung	139 (75.5)	81 (73.6)	58 (78.4)	0.463
Lymph nodes	44 (23.9)	29 (26.4)	15 (20.3)	0.342
Bone	19 (10.3)	12 (10.9)	7 (9.5)	0.751
Liver	15 (8.2)	12 (10.9)	3 (4.1)	0.096
Others	13 (7.1)	7 (6.4)	6 (8.1)	0.651
MSKCC grade				0.598
Favorable	86 (46.7)	49 (44.6)	37 (50.0)	
Intermediate	73 (39.7)	46 (41.8)	27 (36.5)	
Poor	25 (13.6)	15 (13.6)	10 (13.5)	
IMDC risk				0.199
Good	100 (54.3)	57 (51.8)	43 (58.1)	
Intermediate	73 (39.7)	44 (40.0)	29 (39.2)	
Poor	11 (6.0)	9 (8.2)	2 (2.7)	

*MSKCC* Memorial Sloan-Kettering Cancer Center, *IMDC* International Metastatic Renal Cell Carcinoma Database Consortium

### OS and PFS

**Fig. 1:**
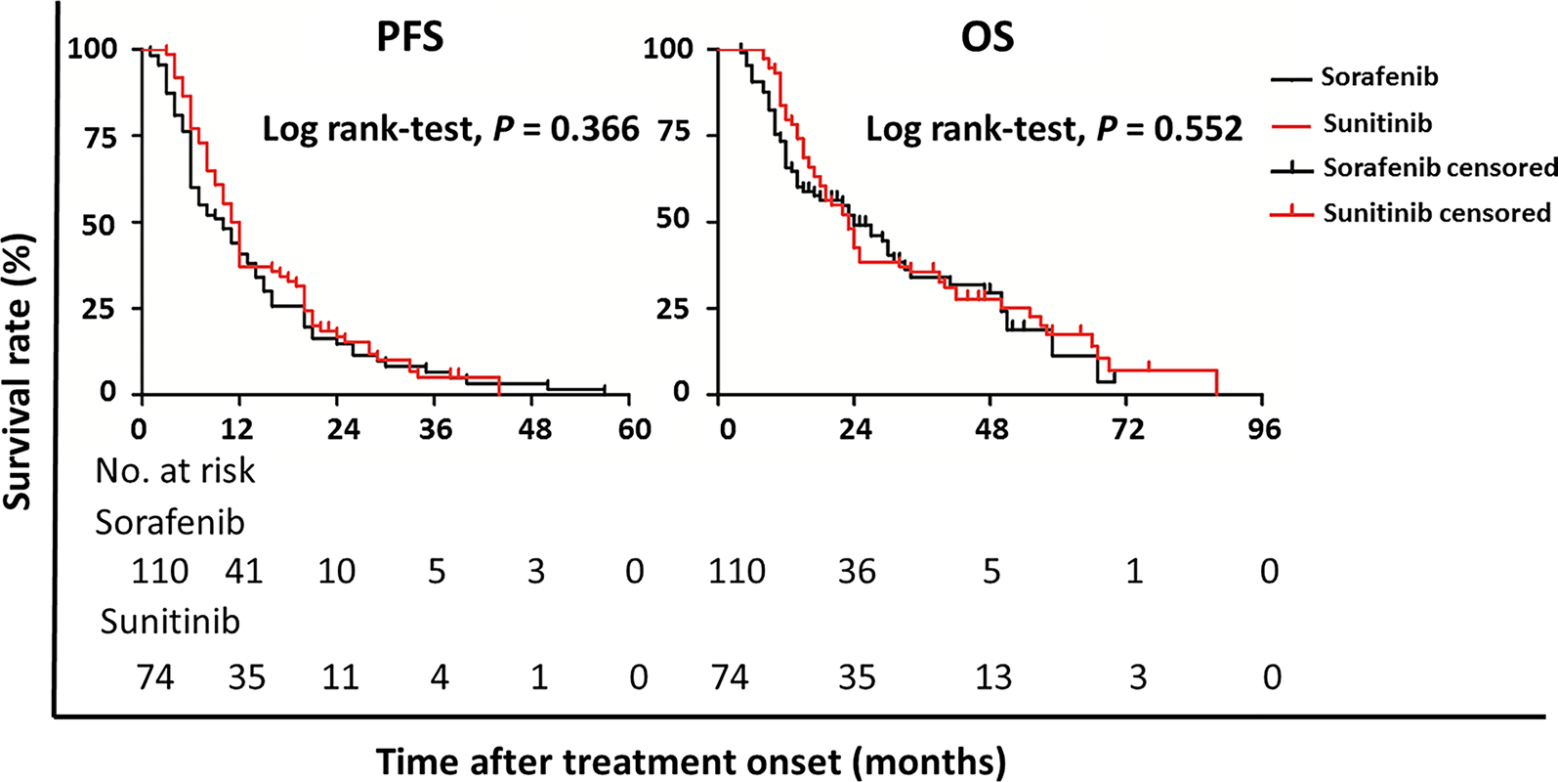
Kaplan–Meier curves of progression-free survival (PFS) and overall survival (OS) of patients with metastatic renal cell carcinoma treated with sorafenib and sunitinib. No significant differences in both PFS and OS were observed between the two groups

Kaplan–Meier curves of PFS and OS are illustrated in Fig. 1. With a median follow-up of 23 months (95% confidence interval [CI] 18–29 months), the median PFS and OS of all the 184 patients were 11 months (95% CI 8–12 months) and 23 months (95% CI 19–27 months), respectively. The median PFS and OS were 10 months (95% CI 7–13 months) and 24 months (95% CI 15–31 months), respectively, in the sorafenib group, which did not significantly differ from the median PFS (11.5 months; 95% CI 9–12 months; *P* = 0.366) and OS (23 months; 95% CI 18–25 months; *P* = 0.552) in the sunitinib group.

### Adverse events

The comparison of AEs between the sorafenib and sunitinib groups is summarized in Table 2. The five most common AEs after treatment onset were hand-foot syndrome (67.3%), diarrhea (62.7%), fatigue (38.2%), nausea (37.3%), and hypertension (20.9%) in the sorafenib group and were hand–foot syndrome (59.5%), fatigue (44.6%), thrombocytopenia (40.5%), nausea (39.2%), and leukocytopenia (36.5%) in the sunitinib group. The treatments were well tolerated with few grade 1–2 AEs. The 3 most common grade 3–4 AEs were hand–foot syndrome (10.8%), diarrhea (1.8%), and anemia (1.8%) in the sorafenib group and were diarrhea (4.1%), leukocytopenia (4.1%), and hypertension (3.6%) in the sunitinib group. In the sorafenib group, 11 (10.0%) patients required dose reduction. In the sunitinib group, 9 (12.2%) patients required dose reduction.

**Table 2:** Comparison of adverse events in the sorafenib and sunitinib groups

Adverse event	Sorafenib group [cases (%)]			Sunitinib group [cases (%)]			*P* value*
	All grade	Grade 1–2	Grade 3–4	All grade	Grade 1–2	Grade 3–4	
Hypertension	23 (20.9)	22 (20.0)	1 (0.9)	23 (36.0)	20 (32.4)	3 (3.6)	0.050
Hand–foot syndrome	74 (67.3)	66 (60.0)	8 (7.3)	44 (59.5)	44 (59.5)	0 (0.0)	0.279
Diarrhea	69 (62.7)	67 (60.9)	2 (1.8)	26 (35.2)	23 (31.1)	3 (4.1)	<0.001
Nausea	41 (37.3)	41 (37.3)	0 (0.0)	29 (39.2)	29 (39.2)	0 (0.0)	0.793
Fatigue	42 (38.2)	42 (38.2)	0 (0.0)	33 (44.6)	33 (44.6)	0 (0.0)	0.385
Alopecia	9 (8.2)	9 (8.2)	0 (0.0)	7 (9.5)	7 (9.5)	0 (0.0)	0.763
Leukocytopenia	12 (10.9)	12 (10.9)	0 (0.0)	27 (36.5)	24 (32.4)	3 (4.1)	<0.001
Anemia	20 (18.2)	18 (16.4)	2 (1.8)	9 (12.2)	9 (12.2)	0 (0.0)	0.272
Thrombocytopenia	12 (10.9)	12 (10.9)	0 (0.0)	30 (40.5)	28 (37.8)	2 (2.7)	<0.001
Hypothyroidism	4 (3.6)	4 (3.6)	0 (0.0)	13 (17.6)	13 (17.6)	0 (0.0)	0.001
Elevation of ALT	11 (10.0)	10 (9.1)	1 (0.9)	10 (13.5)	10 (13.5)	0 (0.0)	0.462

*ALT* alanine aminotransferase

* Grade 1–2 and grade 3–4 toxicities were combined for the comparison

Diarrhea was more common in the sorafenib group than in the sunitinib group (66.4% vs. 31.1%, *P* < 0.001), whereas higher rates of hematologic toxicities such as leukocytopenia (32.4% vs. 10.9%, *P* < 0.001), thrombocytopenia (37.8% vs. 10.9%, *P* < 0.001), and hypothyroidism (17.6% vs. 3.6%, *P* = 0.001) were observed in the sunitinib group than in the sorafenib group.

### QoL

The baseline and post-treatment SF-36 scores did not significantly differ between the two groups (Table 3). There was no significant difference in mean SF-36 scores at all the 8 dimensions between the sorafenib and sunitinib groups at both baseline and 3 months after treatment.

**Table 3:** Quality of life of mRCC patients at baseline and 3 months after treatment with sorafenib and sunitinib

SF-36 dimension	At baseline (score)		*P* value	Three months after treatment (score)		*P* value
	Sorafenib group (*n* = 110)	Sunitinib group (*n* = 74)		Sorafenib group (*n* = 110)	Sunitinib group (*n* = 74)	
PF	68.9 ± 20.9	67.8 ± 19.5	0.548	66.7 ± 24.0	67.1 ± 20.3	0.914
RP	38.2 ± 36.6	45.6 ± 34.2	0.167	36.5 ± 37.5	42.7 ± 40.1	0.527
BP	77.2 ± 16.0	78.7 ± 20.2	0.587	76.2 ± 16.7	77.4 ± 19.7	0.646
GH	56.0 ± 16.3	54.4 ± 20.2	0.531	54.7 ± 15.1	50.8 ± 19.4	0.152
VT	70.1 ± 20.6	71.0 ± 16.1	0.759	68.7 ± 21.7	69.3 ± 15.8	0.858
SF	80.5 ± 18.6	82.1 ± 15.7	0.531	78.5 ± 19.2	80.2 ± 17.3	0.571
RE	59.1 ± 6.3	63.1 ± 33.4	0.452	55.5 ± 39.7	58.6 ± 37.4	0.594
MH	71.1 ± 12.5	75.2 ± 9.8	0.050	69.7 ± 15.7	75.8 ± 14.4	0.055

*PF* physical functioning, *RP* role-physical, *BP* bodily pain, *GH* general health, *VT* vitality, *SF* social functioning, *RE* role-emotional, *MH* mental health

Univariate analysis indicated that the occurrence of grade 3–4 AEs were associated with decreased role-physical score (*P* = 0.013) and bodily pain score (*P* = 0.003) (Table 4). The associations remained significant in multivariate analyses (*P* = 0.017 and 0.005) (Table 5).

**Table 4:** Univariate analysis of associations between clinical characteristics and SF-36 scores of mRCC patients

Variable	PF score		RP score		BP score		GH score		VT score		SF score		RE score		MH score	
	HR (95% CI)	*P* value	HR (95% CI)	*P* value	HR (95% CI)	*P* value	HR (95% CI)	*P* value	HR (95% CI)	*P* value	HR (95% CI)	*P* value	HR (95% CI)	*P* value	HR (95% CI)	*P* value
Sex (man vs. woman)	1.690 (0.832–3.434)	0.147	1.384 (0.698–2.744)	0.353	0.750 (0.354–1.591)	0.453	0.879 (0.425–1.814)	0.987	0.793 (0.400–1.572)	0.507	0.976 (0.493–1.932)	0.944	1.709 (0.823–3.552)	0.151	0.857 (0.433–1.699)	0.659
Age (<65 vs. ≥65 years)	0.838 (0.431–1.631)	0.604	0.962 (0.493–1.879)	0.910	0.764 (0.364–1.607)	0.479	1.020 (0.503–2.067)	0.438	1.830 (0.926–3.618)	0.082	0.982 (0.505–1.910)	0.957	1.151 (0.585–2.266)	0.684	1.412 (0.724–2.751)	0.311
MSKCC grade (favorable vs. others)	1.432 (0.609–3.364)	0.410	1.646 (0.707–3.833)	0.248	0.850 (0.335–2.155)	0.732	1.717 (1.132–4.028)	0.034	0.881 (0.381–2.040)	0.768	1.646 (0.701–3.868)	0.253	1.475 (0.576–3.284)	0.474	1.062 (0.458–2.459)	0.899
IMDC risk (good vs. others)	0.442 (0.465–5.794)	0.442	0.436 (0.112–1.695)	0.231	0.478 (0.100–2.283)	0.355	1.171 (0.330–4.153)	0.807	0.536 (0.152–1.892)	0.322	0.851 (0.251–2.887)	0.796	1.321 (0.374–4.667)	0.666	1.395 (0.411–4.733)	0.593
AE grade (1–2 vs. 3–4)	0.802 (0.312–2.074)	0.804	3.845 (1.327–11.139)	0.013	4.513 (1.689–12.127)	0.003	1.544 (0.589–4.047)	0.377	0.860 (0.334–2.219)	0.756	1.876 (0.706–4.985)	0.207	2.234 (0.772–6.469)	0.138	2.715 (0.987–7.466)	0.053
Treatment (sorafenib vs. sunitinib)	1.199 (0.662–2.173)	0.550	1.096 (0.607–1.977)	0.761	1.340 (0.718–2.504)	0.358	0.617 (0.327–1.164)	0.136	0.733 (0.406–1.325)	0.304	1.074 (0.596–1.938)	0.811	1.377 (0.749–2.531)	0.303	1.243 (0.689–2.243)	0.470

*PF* physical functioning, *RP* role-physical, *BP* bodily pain, *GH* general health, *VT* vitality, *SF* social functioning, *RE* role-emotional, *MH* mental health, *IMDC* International Metastatic Renal Cell Carcinoma Database Consortium

**Table 5:** Multivariate analyses of associations between clinical characteristics and SF-36 scores of mRCC patients

Variable	PF score		RP score		BP score		GH score		VT score		SF score		RE score		MH score	
	HR (95% CI)	*P* value	HR (95% CI)	*P* value	HR (95% CI)	*P* value	HR (95% CI)	*P* value	HR (95% CI)	*P* value	HR (95% CI)	*P* value	HR (95% CI)	*P* value	HR (95% CI)	*P* value
Sex (man vs. woman)	1.611 (0.786–3.302)	0.193	1.419 (0.692–2.909)	0.339	0.737 (0.337–1.613)	0.446	0.884 (0.421–1.885)	0.744	0.861 (0.428–1.730)	0.674	0.955 (0.476–1.917)	0.898	1.673 (0.796–3.513)	0.174	0.850 (0.423–1.710)	0.649
Age (<65 vs. ≥65 years)	0.689 (0.347–1.402)	0.312	0.821 (0.401–1.680)	0.589	0.721 (0.329–1.580)	0.414	0.794 (0.378–1.665)	0.541	1.636 (0.802–3.337)	0.176	0.814 (0.406–1.632)	0.562	1.033 (0.506–2.109)	0.929	1.310 (0.61–2.636)	0.449
MSKCC grade (favorable vs. others)	1.184 (0.441–3.179	0.738	2.557 (0.905–7.339)	0.076	1.089 (0.371–3.195)	0.877	1.927 (0.721–5.153)	0.191	0.887 (0.334–2.357)	0.811	1.939 (0.714–5.265)	0.194	1.128 (0.413–3.085)	0.814	0.804 (0.305–2.122)	0.660
IMDC risk (good vs. others)	0.689 (0.347–1.402)	0.713	0.202 (0.041–1.003)	0.050	0.465 (0.371–3.195)	0.399	0.665 (0.157–2.809)	0.579	0.454 (0.109–1.887)	0.277	0.527 (0.129–2.157)	0.373	1.077 (0.258–4.499)	0.919	1.529 (0.383–6.104)	0.548
AE grade (1–2 vs. 3–4)	0.699 (0.268–1.828)	0.465	3.711 (1.263–10.910)	0.017	4.259 (1.561–11.622)	0.005	1.411 (0.532–3.747)	0.489	0.758 (0.289–1.989)	0.573	1.718 (0.641–4.603)	0.282	2.035 (0.695–5.960)	0.329	2.465 (0.889–6.835)	0.083
Treatment (sorafenib vs. sunitinib)	1.141 (0.620–2.099)	0.671	1.001 (0.539–1.859)	0.998	1.335 (0.694–2.568)	0.387	0.595 (0.311–1.139	0.117	0.735 (0.401–1.348)	0.320	1.033 (0.564–1.890)	0.917	1.364 (0.731–2.545)	0.721	1.340 (0.730–3.462)	0.345

*PF* physical functioning, *RP* role-physical, *BP* bodily pain, *GH* general health, *VT* vitality, *SF* social functioning, *RE* role-emotional, *MH* mental health, *IMDC* International Metastatic Renal Cell Carcinoma Database Consortium

## Discussion

In the present study, we found that sorafenib has comparable efficacy and lower toxicity than sunitinib as first-line therapy for mRCC. AEs were associated with QoL impairments at the role-physical and bodily pain dimensions.

In the present study, the median PFS were 10 months in the sorafenib group and 11.5 months in the sunitinib group (*P* = 0.366). Our findings were similar to those from a study conducted in Korea by Park et al. [14]. In our study, the median OS were 24 months in the sorafenib group and 23 months in the sunitinib group (*P* = 0.552). Our data of OS were shorter than those from the Sheng et al. [15] study, but similar to those from the Park et al. [14] study, and proved that both sorafenib and sunitinib are equally effective as first-line treatment of mRCC in Chinese patients.

In addition to efficacy, toxicities of TKIs are significant factors to be considered while prescribing sorafenib and sunitinib. Randomized clinical trials reported that severe or grade 3–4 toxicities occurred in one-third of the patients treated with sorafenib [7] and two-thirds of the patients treated with sunitinib [21]. Hematologic toxicities are more common with sunitinib than sorafenib in our study. However, the severity of hematologic toxicities could be reduced with discontinuation of sunitinib [21, 22]. Increased risks of bleeding and poor healing are also associated with sunitinib [23], which may severely affect the QoL of patients. In our study, the rates of hematologic toxicities such as leukocytopenia (*P* < 0.001) and thrombocytopenia (*P* < 0.001) were significantly higher in the sunitinib group than in the sorafenib group, which were consistent with the results of previous studies [14, 15]. Hypothyroidism was also more common in the sunitinib group (*P* = 0.001), whereas diarrhea was more common in the sorafenib group (*P* < 0.001). The rates of TKI-related AEs vary among different ethnicities. Ye et al. [6] indicated that Chinese patients were more likely to experience hand-foot syndrome with sorafenib treatment than Western patients, and the occurrence rate in our center was 67.3%. A study in Japan showed that the most frequent AE was elevated lipase followed by hand-foot syndrome and that 10.7% patients had serious AEs [9]. The TARGET study conducted in a Western population showed that diarrhea, rash, fatigue, and hand-foot syndrome were common AEs and hypertension and cardiac ischemia were serious AEs in patients receiving sorafenib treatment [7]. Toxicities of TKIs are also associated with the patient nutritional status. For example, Antoun et al. [24] reported that a low body mass index could be a predictor for a high rate of AEs in patients with mRCC treated with sorafenib. Thus, diverse patient clinical characteristics may lead to diverse rates of AEs in various studies.

Assessment of the changes in QoL after receiving sorafenib or sunitinib treatment is important. Herrmann et al. [25] reported that pretreatment QoL could be a predictor of overall response and OS. Several studies have reported that QoL was not decreased significantly after sorafenib [26] or sunitinib treatment [27]. Furthermore, a phase II randomized controlled trial reported that patients receiving sorafenib treatment had better QoL assessed using the functional assessment of cancer therapy-kidney symptom index (FKSI) and greater treatment satisfaction assessed using the Treatment Satisfaction Questionnaire for Medication (TSQM) compared with patients receiving interferon-alpha treatment [28]. In our study, we observed no significant differences in mean SF-36 scores at all the 8 dimensions between the sorafenib and sunitinib groups at 3 months after the treatment. In a Japanese population, SF-36 scores before and at 3 months after TKI treatment also showed no significant differences [11]. We further analyzed associations between clinicopathologic characteristics and SF-36 scores. There were no significant associations between SF-36 scores and age, sex, MSKCC grade, IMDC risk, or treatment. However, the grade of AEs (grade 1–2 vs. grade 3–4) was independently associated with QoL at the physical functioning (*P* = 0.017) and bodily pain dimensions (*P* = 0.005). This result suggested that timely management of AEs of targeted therapy may eliminate their impact on QoL. Furthermore, a Japanese study observed that the 2-day on/1-day off dosing schedule of sunitinib significantly improved QoL compared with the 4-week on/2-week off dosing schedule [29]. Hence, comparison of the efficacy of TKIs with different dosing schedules is warranted for further validation.

This study has several limitations. First, this was a retrospective study at a single center with limited number of patients, which may cause potential bias of the inferences. The study population was limited to Chinese patients. In addition, to be eligible for the analysis, patients needed to have survived beyond 3 months so as to complete the SF-36 questionnaire at both baseline and 3 months after treatment. Second, a few patients who were intolerant to the drugs had dose titrations, and some switched to second-line targeted therapy, which might have influenced their QoL and survival. Third, the median follow-up was relatively short.

## Conclusions

We demonstrated that sorafenib and sunitinib, as first-line treatment agents, had comparable efficacy on mRCC. Grade 3–4 AEs of TKIs may impair QoL at role-physical and body pain dimensions. Apart from this, neither of the agents had a negative impact on the overall QoL. Multi-center studies with long-term follow-up are warranted to further validate the findings of the present study.

## Abbreviations

AEs: adverse events
CI: confidence interval
BP: bodily pain
CBC: complete blood count
CT: computed tomography
GH: general health
KPS: Karnofsky performance status
MH: mental health
mRCC: metastatic renal cell carcinoma
MRI: magnetic resonance imaging
MSKCC: Memorial Sloan-Kettering Cancer Centre
OS: overall survival
PF: physical functioning
PFS: progression-free survival
QoL: quality of life
RE: role-emotional
RECIST: response evaluation criteria in solid tumors
RP: role-physical
SD: standard deviation
SF: social functioning
SF-36: 36-Item Short Form Health Survey Questionnaire
TKIs: tyrosine kinase inhibitors
VT: vitality
IMDC: International Metastatic Renal Cell Carcinoma Database Consortium
